# Screening of Croatian Native Grapevine Varieties for Susceptibility to *Plasmopara viticola* Using Leaf Disc Bioassay, Chlorophyll Fluorescence, and Multispectral Imaging

**DOI:** 10.3390/plants10040661

**Published:** 2021-03-30

**Authors:** Petra Štambuk, Iva Šikuten, Darko Preiner, Ana Nimac, Boris Lazarević, Zvjezdana Marković, Edi Maletić, Jasminka Karoglan Kontić, Ivana Tomaz

**Affiliations:** 1 Department of Viticulture and Enology, Faculty of Agriculture, University of Zagreb, Svetošimunska cesta 25, 10000 Zagreb, Croatia; pstambuk@agr.hr (P.Š.); isikuten@agr.hr (I.Š.); zmarkovic@agr.hr (Z.M.); emaletic@agr.hr (E.M.); jkkontic@agr.hr (J.K.K.); itomaz@agr.hr (I.T.); 2 Centre of Excellence for Biodiversity and Molecular Plant Breeding, Svetošimunska cesta 25, 10000 Zagreb, Croatia; animac@agr.hr (A.N.); blazarevic@agr.hr (B.L.); 3 Department of Seed Science and Technology, Faculty of Agriculture, University of Zagreb, Svetošimunska cesta 25, 10000 Zagreb, Croatia; 4 Department of Plant Nutrition, Faculty of Agriculture, University of Zagreb, Svetošimunska cesta 25, 10000 Zagreb, Croatia

**Keywords:** *Vitis vinifera* L., downy mildew, biotic stress, chlorophyll fluorescence, spectral indices, imaging methodology, phenotyping model

## Abstract

In the era of sustainable grapevine production, there is a growing demand to define differences between *Vitis vinifera* varieties in susceptibility to downy mildew. Croatia, as a country with a long tradition of grapevine cultivation, preserves a large number of native grapevine varieties. A leaf disc bioassay has been conducted on 25 of them to define their response to downy mildew, according to the International Organisation of Vine and Wine (OIV) descriptor 452-1, together with the stress response of the leaf discs using chlorophyll fluorescence and multispectral imaging with 11 parameters included. Time points of measurement were as follows: before treatment (T_0_), one day post-inoculation (dpi) (T_1_), two dpi (T_2_), three dpi (T_3_), four dpi (T_4_), six dpi (T_5_), and eight dpi (T_6_). Visible changes in form of developed *Plasmopara viticola* (*P. viticola*) sporulation were evaluated on the seventh day upon inoculation. Results show that methods applied here distinguish varieties of different responses to downy mildew. Based on the results obtained, a phenotyping model in the absence of the pathogen is proposed, which is required to confirm by conducting more extensive research.

## 1. Introduction

Ever since the troubling 19th century for the European viticulture production when powdery mildew (*Erysiphe necator*), downy mildew (*Plasmopara viticola*), and phylloxera (*Daktulosphaira vitifoliae*) were introduced from the American continent, winegrowers have been seeking an efficient method of their suppression [1]. While phylloxera was solved by grafting the traditional European grapevine (*Vitis vinifera* L.) varieties on the American vine as a rootstock, which is one of the most successful biological control of this pest spreading, mildews have still been causing problems in all grapevine growing regions around the world, especially with temperate-humid climates [2]. After discovering the fungicide activity of sulphur and copper, and later other active substances, their application became widely used in enormous amounts whose impact on the environment, animal, and human health is harmful [3].

Downy mildew is one of the major grapevine diseases which is caused by an obligate biotrophic oomycete *Plasmopara viticola*, meaning that it uses water and nutrients from its living host plant [4]. The nature of this microorganism is polycyclic and demands temperature in the range from 10 to 29 °C (optimum from 20 to 22 °C) and high humidity (>90%). During winter, it survives in decaying leaves and twigs on the vineyard floor in the form of thick-walled oospores [5]. In spring, when the temperature rises and rains more often, a sporangium is produced from the oospores. Essentially in a drop, two-flagellated zoospores are released from sporangium. They encyst near stoma and the germ tube penetrates inside a green tissue. The mycelium is developed intercellularly in the mesophyll of the grapevine leaves with globose haustoria that invade the cells as the source of *P. viticola* nutrients. When the leaf is infected, yellow-brownish lesions (“oil spots”) develop on its adaxial surface, while sporangia are produced on its abaxial surface and other green tissues such as inflorescences, berries, and tendrils [6]. Sporangia, looking similar to a white cotton cover, are dispersed by wind or rain splash and, as such, are a source of secondary infection cycles. According to Gobbin et al. 2005 [7], there is a continuous input of new genotypes into an epidemic.

During the last century, a lot of efforts have been made in breeding resistant grape varieties by interspecific hybridisation. As a result, cultivars such as Regent in Germany and Bianca in Hungary are auspiciously introduced into the market, together with few dozen newly bred cultivars [8]. Production of resistant cultivars during the last 20 years has been supported with marker-assisted selection (MAS) and carefully designed phenotyping methods. They allow the creation of varieties with higher and more durable resistance [9].

Resistance is a quantitative trait, and quantitative trait loci (QTLs) of resistance to mildews are generally found in non-vinifera germplasm. Loci of resistance to downy mildew are found in *Muscadinia rotundifolia* (*Rpv1*, *Rpv2*) [10], *Vitis rupestris* (*Rpv3*) [11], *Vitis riparia* (*Rpv5*, *Rpv6* [12], *Rpv9* [13]), and *Vitis amurensis* (*Rpv8* [14], *Rpv10* [15], and *Rpv12* [16]). *Muscadinia rotundifolia* is also resistant to powdery mildew, containing loci *Run1* [17,18] and *Run2* [19]. However, locus containing resistance to powdery mildew, specifically *Ren1*, is found in two cultivars originating from central Asia, Kishmish vatkana, and Dzhandzhal kara (*V. vinifera*) [20]. Cultivars Regent and Solaris are highly resistant to downy mildew, and their typical response to the disease is small brownish spots (necrosis formation). Nevertheless, sporulation emerging from the discoloured tissue indicates that not all cells have undergone programmed cell death [21,22]. The main morphological barrier of *V. riparia* to *P. viticola* attack is the presence of the inner cuticular rim which is a constitutive trait independent of infection [23]. While North American and Asian *Vitis* species develop necrotic spots after *P. viticola* infection, they are not observed in Georgian *Vitis* germplasm, meaning that their defence mechanisms are different [24]. Recently, *V. vinifera* varieties are of great interest to research due to their high genetic variability, local/regional importance, and lacking genetic background with undesirable features. Moreover, differences in susceptibility to downy mildew are found in Spanish [25] and Georgian [26,27] collections between *V. vinifera* varieties.

Since leaves are the pioneers in providing the first visual symptoms of the downy mildew disease, phenotyping methods on leaf discs that are inoculated and maintained in controlled conditions have been widely applied among plant pathologists, breeders, and geneticists who are willing to obtain differences between genotypes regarding their downy mildew susceptibility [28,29]. Leaf disc bioassay is based on the International Organisation of Vine and Wine (OIV) descriptor 452-1 (Leaf: degree of resistance to *Plasmopara* (leaf disc test)) [30]. Leaf disc test is widely accepted and used by many authors [22,31,32,33] whose aim is to distinguish levels of susceptibility to downy mildew between different varieties. When this method is properly performed, it is reliable and useful for predicting each variety’s susceptibility to downy mildew in field conditions [34].

Apart from visible detection of downy mildew infection, there are sophisticated methods that measure plant’s stress levels in form of photosynthesis (in)efficiency. Novel phenotyping methods which include chlorophyll fluorescence and multispectral imaging were previously used for quantification of different plant diseases such as *Blumeria graminis* in barley [35], *Cercospora beticola* in sugar beet [36], and *Puccinia triticina* in wheat [37]. Screening for susceptibility to *P. viticola* among *V. vinifera* varieties was also performed by these methods [38,39]. Recently, alterations of primary metabolism induced by pathogenesis have been the focus of studies. For this purpose, chlorophyll fluorescence imaging, as a non-invasive method, is of principal value since it measures both spatial and temporal changes in photosynthetic processes localised with high precision within plant tissues [40,41]. Generally, downy mildew infection costs energy either for the induction of plant defences or the destruction of carbohydrates. Yellow-brownish lesions (chlorosis) of grapevine photosynthesizing tissues (e.g., leaves) implicate that infection leads to the destruction of chlorophyll and subsequent blockage of CO_2_ fixation processes [41].

Chlorophyll fluorescence measurements are based on three possible ways (outcomes) of photon energy transfer—thermal dissipation (heat), photochemistry, and chlorophyll fluorescence emission. When excitations are neither lost as heat nor lead to photochemistry, they are re-emitted as light in a process called chlorophyll *α* fluorescence [42]. An increase in chlorophyll fluorescence thus implies a decrease in photosynthesis and/or thermal dissipation, and vice versa [43]. It can be used for the early detection of biotic stress, even before the manifestation of visible downy mildew symptoms [38]. As an early answer to downy mildew infection, the plant’s primary and secondary metabolism can be affected due to the initiation of plant defence [44].

In plant phenotyping, the application of imaging spectroscopy came from research on the remote sensing of vegetation [45]. Spectral reflectance information of leaves or canopies is used to quantify vegetation indices, which are ratios and differences between spectral reflectance data at given wavelengths (e.g., near-infrared wavelengths (700–1200 nm)) [46]. These indices have been used for fast, non-destructive measurements of green biomass, chlorophyll content, leave and canopy senescence, and plant water status, which can be applied in both field research and breeding programs for large-scale phenotyping [45]. 

Since Croatia has a long tradition of cultivating grapevine in its geographically and climatically different regions, at least 95 are considered native [47,48] whose susceptibility to main diseases is necessary to define in order to describe their complete biological and economical potential. For this purpose, a study concerning differences in susceptibility to downy mildew was conducted on 25 native grapevine varieties by applying a leaf disc bioassay with chlorophyll fluorescence and multispectral imaging. The aim of this study was (i) to assess the susceptibility among *V. vinifera* varieties to downy mildew by applying leaf disc test, (ii) to examine whether chlorophyll fluorescence and multispectral imaging of leaf discs are suitable methods for distinguishing genotypes of different susceptibility to downy mildew, and (iii) to test the relationship between distinctive OIV classes and their fluorescence and multispectral traits in the absence of the pathogen. 

## 2. Results

### 2.1. Differences in Chlorophyll Fluorescence and Multispectral Imaging Responses between Infected and Non-Infected Leaf Discs

**Figure 1:**
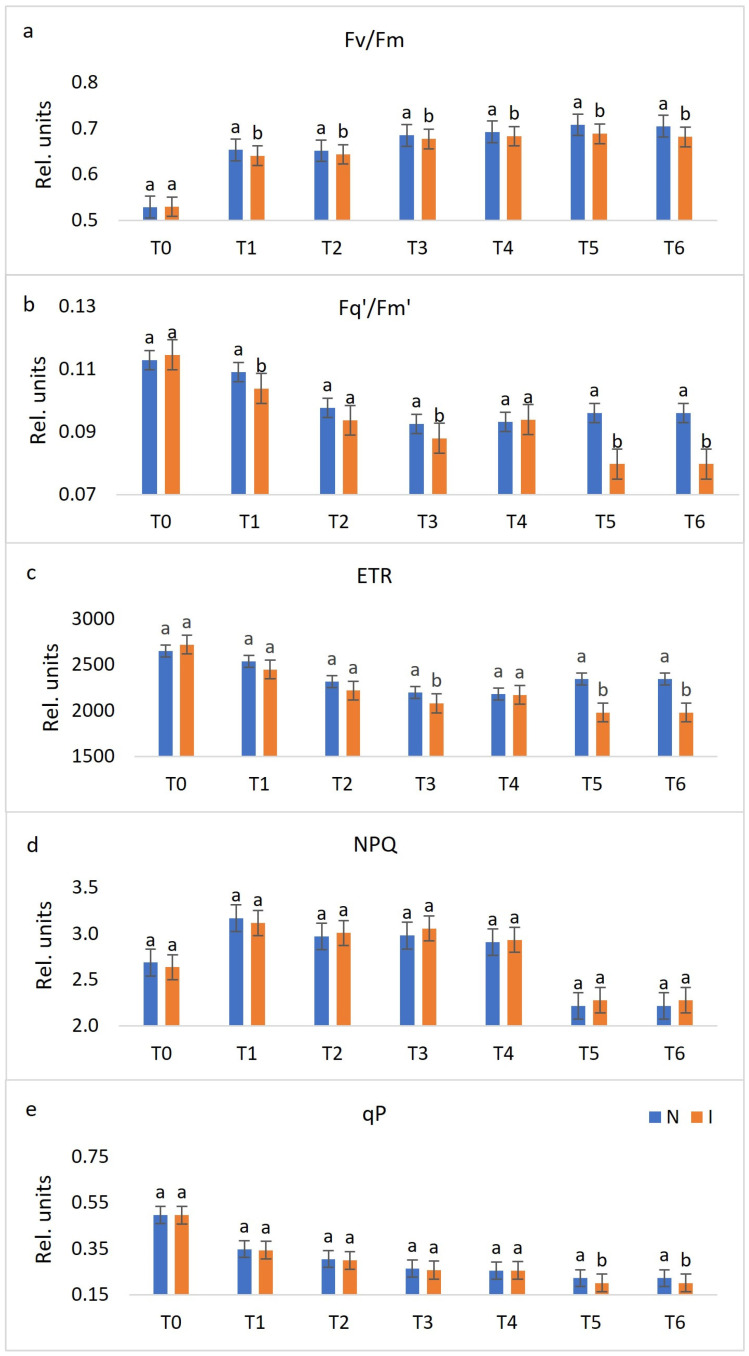
Changes between non-infected (N) and infected (I) leaf discs throughout seven terms (before treatment (T_0_), one day post-inoculation (dpi) (T_1_), two dpi (T_2_), three dpi (T_3_), four dpi (T_4_), six dpi (T_5_) and eight dpi (T_6_)) in chlorophyll fluorescence parameters (the average of 30 genotypes). Differences between the means were evaluated by Duncan’s multiple range test at a confidence level of 95% (*p* < 0.05). Means with the same letter are not significantly different. Sub-figures depict parameters as follows: (**a**) F_v_/F_m_, (**b**) F_q’_/F_m’_, (**c**) ETR, (**d**) NPQ, and (**e**) qP.

**Figure 2:**
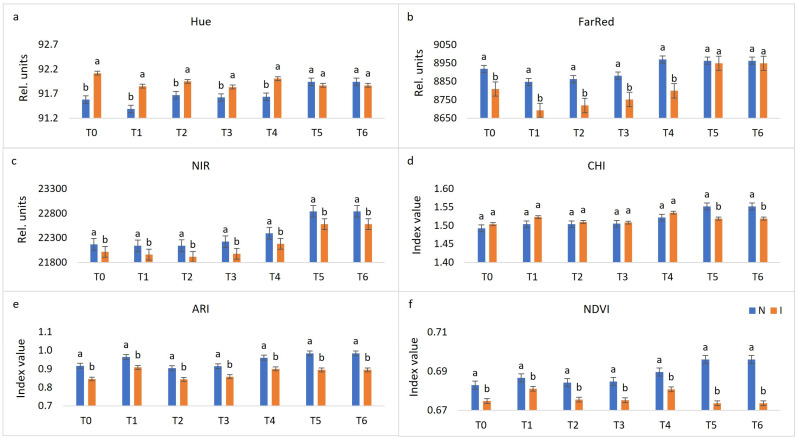
Changes between non-infected (N) and infected (I) leaf discs throughout seven terms (before treatment (T_0_), one day post-inoculation (dpi) (T_1_), two dpi (T_2_), three dpi (T_3_), four dpi (T_4_), six dpi (T_5_) and eight dpi (T_6_)) in multispectral parameters (the average of 30 genotypes). Differences between the means were evaluated by Duncan’s multiple range test at a confidence level of 95% (*p* < 0.05). Means with the same letter are not significantly different. Sub-figures depict parameters as follows: (**a**) Hue, (**b**) FarRed, (**c**) NIR, (**d**) CHI, (**e**) ARI, and (**f**) NDVI.

Chlorophyll fluorescence and multispectral imaging were performed in seven terms, namely, before treatment (T_0_), one day post-inoculation (dpi) (T_1_), two dpi (T_2_), three dpi (T_3_), four dpi (T_4_), six dpi (T_5_) and eight dpi (T_6_). The data presented in Figure 1 and Figure 2 are the average of 30 genotypes included in this research. Visible symptoms in the form of sporulation appeared on the sixth and seventh day after inoculation on the most of evaluated *V. vinifera* varieties and control genotypes Solaris and Regent, respectively. Solaris developed necrotic spots on the fourth day after inoculation, while *V. riparia* showed no visible changes. Imaging started with non-infected grapevine leaf discs and terminated after downy mildew sporulation developed. Evaluated fluorescence parameters included maximum quantum yield of photosystem II (PSII) (F_v_/F_m_), effective quantum yield of photosystem II (PSII) electron transport (F_q’_/F_m’_), electron transport rate (ETR), non-photochemical quenching (NPQ), and photochemical quenching (qP), while the focus of multispectral imaging was on colour appearance parameter (Hue), far-red reflectance (FarRed), near-infrared reflectance (NIR), chlorophyll index (CHI), anthocyanin index (ARI) and normalised difference vegetation index (NDVI). Among these 11 parameters, most were significantly different between non-infected and infected leaf discs in at least two terms of measurement (Figure 1 and Figure 2). However, no significant difference was found for NPQ between these two variants of leaf discs (Figure 1d). A detailed description for each parameter follows below.

F_v_/F_m_ was significantly different in all terms of measurement, except in the pre-infection stage (T_0_) (Figure 1a). From T_1_ to T_6_, non-infected leaf discs reached higher values, compared to infected ones, which is expected since decreasing values of this parameter indicate plant stress [49]. 

The values of F_q’_/F_m’_ showed to be distinctive in T_1_, T_3_, T_5,_ and T_6_, with lower values for infected leaf discs (Figure 1b). Similar is observed for ETR values, although this parameter was not significant in T_1_ (Figure 1c). Their overall change throughout the period of measurement slightly decreased.

The trend of NPQ (Figure 1d) gradually fell from T_1_ to T_6_, while total values of qP decreased during the experiment period. Slightly lower qP values were observed for infected leaf discs, compared to non-infected ones in all terms, although this difference was significant only in the later stages of infection (T_5_ and T_6_) (Figure 1e). In T_0_, values for both variants of leaf discs were 0.5, while the values for infected leaf discs were reduced by more than a half during six and eight days after inoculation.

Multispectral imaging parameters Hue (Figure 2a) and FarRed (Figure 2b) were not significantly different during the final two measurements between non-infected and infected leaf discs, meaning that by these two parameters, it is not possible to distinguish between non-infected and infected leaf discs from the occurrence of visible symptoms. However, from T_0_ until T_4_, significantly higher Hue values were observed for infected leaf discs, while the same is true for non-infected leaf discs as far as FarRed values are concerned.

**Figure 3:**
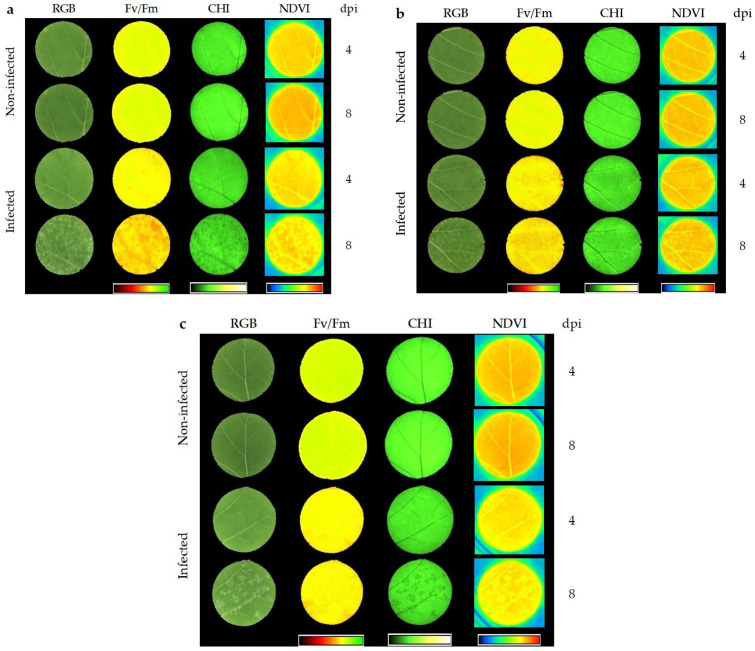
RGB (visual impression), F_v_/F_m_, chlorophyll index (CHI), and normalised difference vegetation index (NDVI) images of (**a**) Lasina (OIV (International Organisation of Vine and Wine) 1), (**b**) Malvasija dubrovačka (OIV 3), and (**c**) Malvazija istarska (OIV 5) taken at four (T_4_) and eight (T_6_) dpi.

NIR (Figure 2c), ARI (Figure 2e) and NDVI (Figure 2f) values were statistically different for infected and non-infected leaf discs throughout the whole experiment with higher values for non-infected ones. The values of CHI (Figure 2d) were statistically different during the final two terms, while lower values were observed for infected leaf discs, compared to non-infected ones during this final stage of inoculation. The differences between non-infected and infected leaf discs of parameters NIR and ARI remained almost the same throughout the time of the experiment, while NDVI differences fluctuated from T_0_ until T_4_ and were the highest in the last two terms. Unlike the visible changes that can be observed six or seven days upon inoculation in the form of *P. viticola* sporulation, through fluorescence (F_v_/F_m_) and multispectral (CHI and NDVI) channels, it is possible to differentiate non-inoculated from inoculated leaf discs at 4 dpi (Figure 3).

### 2.2. Differences in P. viticola Sporulation on Leaf Discs among Genotypes 

According to the OIV leaf disc test, on all susceptible *V. vinifera* varieties, *P. viticola* sporulation was developed as expected. However, significant differences in sporulation density and covered surfaces were determined between different varieties. Thus, they were grouped in separated OIV classes, as shown in Table 1, while examples of different visible phenotypic reactions and corresponding OIV classes are presented in Table 2.

### 2.3. Differences in Chlorophyll Fluorescence and Multispectral Imaging Responses between Diverse OIV Classes

**Figure 4:**
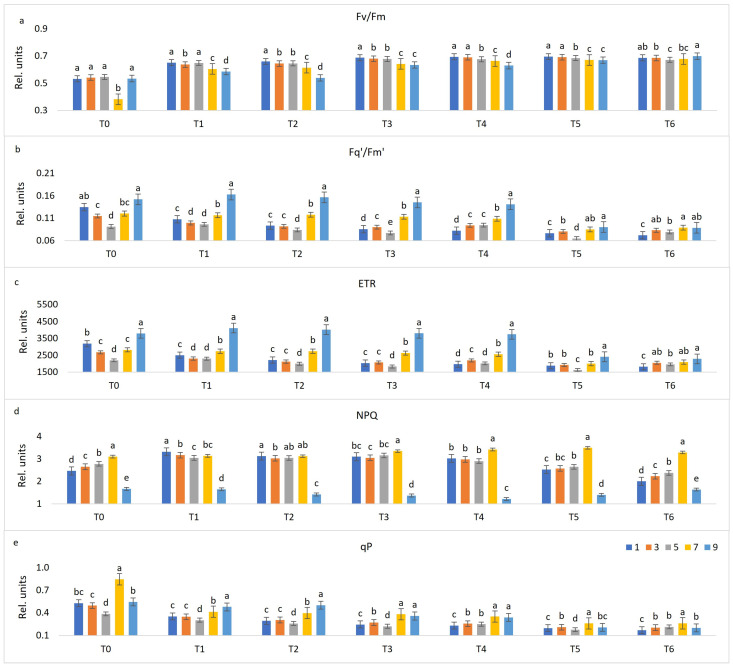
Changes between distinctive OIV classes (1—most susceptible; 9—resistant) throughout seven terms (before treatment (T_0_), one day post-inoculation (dpi) (T_1_), two dpi (T_2_), three dpi (T_3_), four dpi (T_4_), six dpi (T_5_) and eight dpi (T_6_)) in chlorophyll fluorescence parameters. Differences between the means were evaluated by Duncan’s multiple range test at a confidence level of 95% (*p* < 0.05). Means with the same letter are not significantly different. Sub-figures depict parameters as follows: (**a**) F_v_/F_m_, (**b**) F_q’_/F_m’_, (**c**) ETR, (**d**) NPQ, and (**e**) qP.

The distinctiveness of OIV classes was shown to be significant in specific terms and by specific parameters. An overall slight increase of F_v_/F_m_ (Figure 4a) values was noticed through the terms. In T_2_, T_3_, T_4,_ and T_5_ a downward trend is observed from the OIV most susceptible group of genotypes to the resistant group. The highest distinctiveness of the OIV classes was found four days upon inoculation (T_4_) when no significant difference was found only between classes 1 and 3.

Another important indicator of a plant’s biotic stress is F_q’_/F_m’_ (Figure 4b). In contrast to F_v_/F_m_, an overall slight reduction of average F_q’_/F_m’_ values throughout the measurement period was observed. The OIV classes 9 (*V. riparia*) and 7 (Regent and Solaris) had the highest values in comparison to other OIV classes in T_1_, T_2_, T_3,_ and T_4_. Similar to F_v_/F_m_ responses in T_4_, the separation of different OIV classes was highly distinctive, although classes 3 and 5 were not significantly different in this term. The least distinctiveness was observed in T_5_ and T_6_ with two and one significantly different OIV classes, respectively.

Similar results were obtained for F_q’_/F_m’_ (Figure 4b) and ETR (Figure 4c). The highest and significantly different ETR values were observed for the OIV class 9 in each term with the exception of the last term when it was not statistically different from the OIV classes 3 and 7. In T_2_ and T_3_, the OIV classes 1 and 3 were not statistically different, while in T_1_, the same was true for the OIV classes 3 and 5. At later stages (T_5_ and T_6_) of infection, averaged values of parameters F_q’_/F_m’_ (Figure 4b) and ETR (Figure 4c) among all OIV classes declined.

Differences in NPQ among OIV groups through seven terms are depicted in Figure 4d. Significantly the lowest values were ascribed to the OIV class 9, while its neighbouring class 7 had the highest values in each term, except T_1_ and T_2_. A gradual decline can be observed in average values for each term from one day upon inoculation (T_1_) to eight days upon inoculation (T_6_). In T_4_, there was no statistical difference between susceptible OIV classes 1, 3, and 5. It is interesting to notice the similarity of the bar charts depicting the pre-infection stage (T_0_) and the final stage (T_6_). In both terms, each OIV class is significantly different from the others. Increasing values can be found from class 1 to class 7, while class 9 had the lowest value as abovementioned.

As an indicator of opened PSII reaction centres [50], qP decreased throughout the period of imaging (Figure 4e). The same trend was noticed in T_1_ and T_2_ with no significant difference between classes 1 and 3, whereas increasing and significantly different values are observed from classes 5 to 9. In T_6_, classes 3, 5, and 9 were not significantly different.

**Figure 5:**
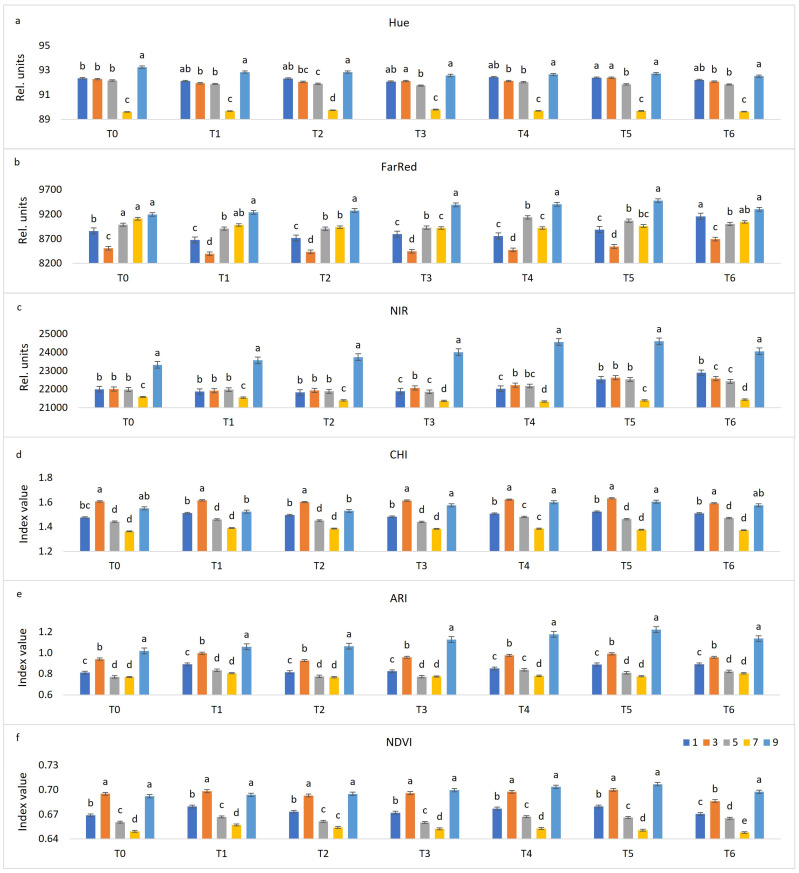
Changes between distinctive OIV classes (1—most susceptible; 9—resistant) throughout seven terms (before treatment (T_0_), one day post-inoculation (dpi) (T_1_), two dpi (T_2_), three dpi (T_3_), four dpi (T_4_), six dpi (T_5_) and eight dpi (T_6_)) in multispectral parameters. Differences between the means were evaluated by Duncan’s multiple range test at a confidence level of 95% (*p* < 0.05). Means with the same letter are not significantly different. Sub-figures depict parameters as follows: (**a**) Hue, (**b**) FarRed, (**c**) NIR, (**d**) CHI, (**e**) ARI, and (**f**) NDVI.

Hue values fluctuated between the terms of imaging, while the highest overall value of all OIV classes was observed in T_1_ (data not shown). In each term, class 7 had the lowest values, while there were no considerable differences between other classes (Figure 5a).

An upward trend from T_1_ to T_6_ is observed for far-red fluorescence values of all infected leaf discs (data not shown). Moreover, a slightly increasing trend was observed in T_0_, T_1_, T_2,_ and T_6_ from class 5 to class 9 (Figure 5b). Significantly the lowest values are measured for class 3 in each term.

The values of NIR (Figure 5c) were higher in the later stages of downy mildew development, compared to the early stages. In all terms, class 9 reached the highest values, while its neighbouring class 7 had the lowest values. Classes susceptible to downy mildew (1, 3, and 5) showed similar values of this parameter.

The values of CHI (Figure 5d) and ARI (Figure 5e) showed no significant differences in all terms between classes 5 and 7, except for ARI in T4. Both indices reached their highest values in T_4_ and T_5_. Generally, similar bar charts are obtained for these indices, and for NDVI (Figure 5f), with the highest values for classes 3 and 9, and the lowest for 5 and 7 in all terms of imaging. For NDVI, there was no statistical difference between classes 3 and 9 in all terms, except in the final term, in which class 9 reached statistically higher values.

More pronounced differences between OIV classes can be observed by parameters of chlorophyll fluorescence (F_v_/F_m_, F_q’_/F_m’_, ETR, NPQ, and qP) during the first five terms of imaging, while at the end of the trial, the values for most of them decreased and became more unified. However, the parameters of multispectral analysis (Hue, FarRed, NIR, CHI, ARI, NDVI) mostly followed the same pattern from T_0_ until T_6_, which led to the conclusion to propose a phenotyping model for differentiation of OIV classes in the absence of *P. viticola* using the results obtained in T_0_.

### 2.4. Phenotyping Model

One of the objectives of the present study was to find a reliable phenotyping model of grapevines’ susceptibility to downy mildew. After comparing the final OIV grouping made by different sporulation on leaf discs with responses of chlorophyll fluorescence and multispectral imaging in the pre-infection term (T_0_), and their interactions, a relationship was determined using the logistic regression. The training set of this model consisted of 19 genotypes and 11 measured parameters described in Table 4. This set included 15 Croatian native varieties and two susceptible international *V. vinifera* L. varieties (Cabernet Sauvignon, Chardonnay) to model three susceptible OIV classes (1, 3, and 5), while Solaris and Regent were used to form the OIV class 7 that represents almost completely resistant genotypes. Three genotypes (Cabernet Sauvignon, Divjaka, and Malvazija istarska) were used in two repetitions while the rest of the genotypes were used once. The OIV classes 1, 3, 5, and 7 were represented with 4, 6, 10, and 2 observations, respectively. All of them were classified to the training set with complete accuracy. High R^2^ was obtained between the measured parameters and the OIV classes with a value of 0.92 for Cox and Snell’s R^2^, and values close to 1 for McFadden’s and Nagelkerke’s R^2^. Accordingly, the log-likelihood value for each observation was close to 0. 

The prediction set consisted of 10 Croatian native varieties that possess different levels of susceptibility to downy mildew according to the OIV leaf disc test. Interestingly, half of the varieties that were the most susceptible and produced the densest sporulation on the leaf discs (the OIV class 1) belonged to the same class according to the given model. However, the other half was grouped to the neighbouring class 3. A similar observation was obtained for varieties from class 3. The model put half of them to the same class, while the rest varieties were dispersed to the neighbouring classes 1 and 5. Finally, the model was 100% correct for class 5. None of the varieties from the prediction set was put in class 7 provided by the training set, which confirmed the strength of the model (Table 3).

## 3. Discussion

Leaf discs test proved to be a simple method to perform and provide results about differences in susceptibility to downy mildew in a short time (usually not longer than seven days) starting from inoculation until the development of *P. viticola* sporulation. To our knowledge, research that includes a high number (25) of Croatian native varieties by using this method was conducted for the first time. Interesting and trustworthy results are gained since genotypes with a known level of susceptibility or resistance were comparatively evaluated. Previously, other authors [21,29,31,51,52,53] likewise included Solaris, Regent, *V. riparia*, Chardonnay, and Cabernet Sauvignon in their studies with leaf discs, although their scales for determining the level of susceptibility slightly differ one from another. Nonetheless, all these results are similar (or the same) and comparable. More precisely, Regent was characterised as resistant (class > 7) [31] and partially resistant [21,29,51], whereas Solaris was partially resistant [21,29]. Regarding the North American species, *V*. *riparia* was confirmed as the most resistant species, followed by *V*. *aestivalis* and *V. rupestris*. *V. riparia* allowed no sporulation and seldom showed necrotic spots [51], which is in agreement with the present research. Coevolving on the same continent with downy mildew, these North American species were subjected to the same stressful stimulus and gained epigenetic modifications responsible for their defence systems [52]. Differences among cultivars in response to the action of *P. viticola* are related to different passive mechanisms (i.e., dense hydrophobic trichomes on the abaxial side of leaves) and active responses involving hypersensitivity and synthesis of specific secondary metabolites [1,5]. Chardonnay was classified as the most susceptible genotype, and *V. riparia* was highly resistant in experiments conducted by [53]. Cabernet Sauvignon was described as a little susceptible cultivar, together with Riesling, Pinot Noir, and Pinot Blanc [54], which could be assigned to the OIV class 5, where Cabernet Sauvignon belongs by here presented results. 

However, this type of phenotyping relies largely on visual scoring, which is time-consuming especially for large-scale experiments. Moreover, it can generate bias between different experts and experimental repeats. Due to the rapid development of high-throughput genotype screening in plant breeding and genomics, there is a call for more effective and reliable phenotyping data to support modern genetic crop improvement [45]. For that reason, the leaf disc test was complemented with chlorophyll fluorescence and multispectral imaging in this research to describe differences between distinctive OIV groups and changes between non-infected and infected leaf discs.

Photosynthesis is one of the most important processes of a plant’s primary metabolism, meaning that its inhibition is one of the first signals of plant stress. It serves as a plant defence mechanism against biotic stress by limiting the nutrient availability to the pathogens. On the other hand, pathogens are able to manipulate the plant metabolism for their own benefit [55]. The most sensitive chlorophyll fluorescence parameters of grapevine leaves being infected with *P. viticola* are F_v_/F_m_ and F_q’_/F_m’_ [38]. The decreases in the F_v_/F_m_ ratio (variable to the maximum value of chlorophyll *a* fluorescence) indicate the reduction of photosystem II efficiency, specifically photoinhibition [56]. Photoinhibition is a phenomenon resulting from a reduction of photosynthetic activity predominantly due to light-induced decreases in CO_2_ assimilation [57].

According to previous studies [58,59], an optimal value of F_v_/F_m_ is 0.83 for most plant species, while values lower than this mean that the plant is exposed to stress and its photosynthetic performance is impaired. These findings can be ascribed to overall low F_v_/F_m_ values (< 0.71) obtained in the present study, because of *P. viticola* infection and due to conducting the experiment on excised leaf parts and imaging their abaxial sides. The lowest values of F_v_/F_m_ observed in T_0_ are probably the result of leaves cutting and their changing environment from the greenhouse to the laboratory, where leaf discs were placed on wet filter papers. Despite these circumstances, only 24 h after inoculation did this parameter clearly distinguish infected from non-infected leaf discs (Figure 1a), which is much earlier than the previous finding where the earliest change of F_v_/F_m_ pattern on Chardonnay leaves appeared four days upon inoculation [38]. Here, necrotic areas were observed four days after inoculation in cultivar Solaris, which is in accordance with previous research [39], in which low F_v_/F_m_ value was found five days after inoculation due to the development of necrotic spots. 

On the contrary, F_q’_/F_m’_ and ETR values were generally lower for infected susceptible *V. vinifera* varieties (OIV classes 1, 3, and 5) compared to infected Solaris, Regent (OIV 7) and *V. riparia* (OIV 9), suggesting that in spite of being infected, these (partially) resistant genotypes keep higher photosynthetic rate. Yet, their performance also declined during the later stage of infection (6 and 8 dpi) (Figure 4b,c). These changes can be explained by gradual chlorophyll degradation [43] and destruction of the photosynthetic apparatus [49] due to both *P. viticola* infection and leaf discs senescing. ETR can be stimulated in regions adjacent to infected cells to provide energy to fuel defence responses or as a result of compensation for loss of green leaf area [49].

NPQ refers to thermal energy dissipation in the PSII antennae [41]. It was previously reported that its values (together with F_q’_/F_m’_) decreased in tomato leaves infected by *B. cinerea* in developing lesions. The surrounding areas were also characterised by decreased NPQ, which is indicative of enhanced ATP consumption on CO_2_ fixation in the Calvin–Benson cycle [60]. By comparing the interaction of powdery mildew with susceptible and resistant lines of barley, the impact in the compatible interaction was much greater, meaning that the greatest reduction in F_q’_/F_m’_ and NPQ in the site of infection that extended to neighbouring cells was observed in susceptible line [35]. In the present study, although NPQ responses were not useful for distinguishing infected from non-infected leaves, its values plunged at 6 dpi in both treatments (Figure 4d) when necrotic spots and sporulation had already been developed in infected tissues. Furthermore, this decline was more pronounced for susceptible OIV classes (1, 3, and 5), compared to resistant classes whose values did not change considerably during the experiment (Figure 4d).

Photochemical quenching (qP) indicates the proportion of PSII reaction centres that are open; thus, a change in qP is due to the closure of reaction centres, resulting from a saturation of photosynthesis by light. This parameter, together with F_v_/F_m_, provides information about the underlying processes which have altered photosynthetic efficiency [50]. A downward trend of photochemical quenching is observed in our study (Figure 1e), in accordance with [61]. This parameter can also be used as a discriminator of susceptible and resistant genotypes until the first appearance of visible changes (4 dpi) because, after that, all groups of genotypes showed similar (and very low) qP values (Figure 4e).

Hue values are proportional to total chlorophyll, offering an alternative to photometric analysis of leaf extracts. This is demonstrated using tobacco leaves with various chlorophyll contents due to senescence and thus shows the possibility of applications in studies of stress conditions accompanied by chlorophyll loss [62]. In this colour space, each colour can be expressed independently from its saturation (pale or intense colour) and value (dark or bright colour). This feature can be used for in-field detection of downy mildew symptoms [63]. In our research, this trait clearly resolved cultivars Solaris and Regent (OIV 7) (Figure 5a) probably due to considerably brighter green colour of their leaves abaxial sides (https://www.vivc.de accessed on 9 February 2021) and subsequent lower hue values from all other evaluated genotypes. Higher FarRed values are mostly observed in genotypes which are more tolerant to downy mildew (Figure 5b) and in non-infected leaf discs (Figure 2b) since the pathogen’s mycelium destroys chloroplasts.

Leaf reflectance is very high in the near-infrared at ~800 nm when leaves are also largely transparent [64]. The absorption by leaf pigments is strongly reduced in this spectrum, and thus, both reflectance and transmittance are much higher than in the visible spectral range. A decrease of the reflectance may be an indicator of reduced areal interspaces (reduced assimilation of CO_2_) in the mesophyll of leaves under stress conditions [40]. For that reason, *V. riparia* showed the highest values in this spectrum as the most resistant evaluated genotype (Figure 5c). It has also been reported that *V. riparia* have smaller, more loosely packed cells with extended intercellular space for the spongy parenchyma [65].

Chlorophyll and anthocyanin contents were calculated by CHI and ARI, respectively. By these measurements, the highest contents of chlorophyll and anthocyanin are observed in the OIV classes 3 and 9 with no considerable changes throughout the measurement period (Figure 5d,e). However, at 6 and 8 dpi, CHI distinguished infected and non-infected leaf discs (Figure 2d). Oerke et al. [21] found decreasing chlorophyll content during disease development which was associated with the appearance of visible symptoms on the adaxial leaf side, such as discolouration and oil spots. NDVI, as an indicator of the plant’s health status, clearly separated inoculated from non-inoculated leaf discs, especially in the later stages of infection (Figure 2f). Visible changes were observed six or seven days upon inoculation in the form of *P. viticola* sporulation, while through fluorescence (F_v_/F_m_) and multispectral (CHI and NDVI) channels was possible to differentiate non-inoculated from inoculated leaf discs at 4 dpi (Figure 3), and these differences are often more pronounced among the genotypes from the OIV class 1 (Figure 3a). The difference between infected and non-infected leaf discs in T0 can be explained by initial differences in plant material, i.e., position and exposure to the light during the development of the leaves. Due to this fact, changes in the difference between infected and non-infected leaf discs throughout seven terms against T0 must also be considered in the case of parameters Hue, FarRed, NIR, ARI, and NDVI. 

Applications of fluorescence imaging in screening for disease and stress resistance have a clear potential for quantitative assessment of the plant infection or stress level before the appearance of visible symptoms [40]. An example is detecting whether an asymptomatic *V. vinifera* variety Malvasía de Banyalbufar is infected by GLRaV-3 (*Grapevine leafroll-associated virus 3*) [66]. It was previously reported that logistic regression analysis enabled the determination of probabilistic leaf–cluster relationship in downy mildew natural infection on Cabernet franc [67].

Preliminary results of the proposed model suggest that by chlorophyll fluorescence and multispectral imaging, it is possible to distinguish grapevine genotypes with different susceptibility to downy mildew even before the conditions for the pathogen development are satisfied and before the grapevine inoculation since this model is formed on non-infected leaf discs. However, it is necessary to confirm the model by conducting a more comprehensive experiment with a greater number of genotypes. Imaging of whole leaves and their adaxial sides with high chlorophyll content in densely packed palisade parenchyma, in contrast to spongy parenchyma on the abaxial side [68], and imaging other susceptible tissues (i.e., inflorescence, green berries, and tendrils), will provide more complete information. Once the model is confirmed, the next step is generating a large-scale data platform by imaging the genotypes with known response to downy mildew to create an explanatory background for linking genotypes to phenotypes. This method could be applicable for high-throughput phenotyping (screening) of seedlings that are the result of breeding programs aiming to create genotypes with high resistance to mildews. In this way, the proper OIV classes could be ascribed to many seedlings at the early stage of their development. Another possible application is the phenotyping of existing grapevine collections and commercial vineyards with no defined differences in susceptibility to downy mildew between different genotypes, which is of utterly importance in the era of sustainable agricultural production and precision viticulture.

## 4. Materials and Methods

### 4.1. Plant Material

Altogether, 30 genotypes were included in this research—25 Croatian native varieties, two susceptible international *V. vinifera* varieties (Cabernet Sauvignon, Chardonnay), two resistant cultivars (Regent, Solaris), and one *Vitis* species (*Vitis ripara*). One-year cuttings, 20 cm long, containing three to four buds were taken from the Croatian native grapevine varieties collection, Department of Viticulture and Enology, University of Zagreb Faculty of Agriculture in March 2019. Before planting, a bud from the basal part of each cutting was removed, and the cuttings were soaked overnight in an aqueous solution containing 0.1 mg L^−1^ indole-3-butyric acid (IBA). Each cutting was planted in a 5 L drip-irrigated pot containing standard commercial substrate S2 (Klasmann-Deilmann, Geeste, Germany). The plants were grown in a greenhouse. Fungicide Chromosul^®^ (Chromos Agro, Zagreb, Croatia) was applied in each season to control powdery mildew infection. This fungicide is sulphur based and only has preventive-contact activity on powdery mildew; nevertheless, young leaves sampled at the stage of 10 fully developed leaves were not treated. Each genotype was represented by 12 cuttings. In 2020 shoots’ development was uniformed. When they reached a growing stage of 10 fully developed leaves, the fourth and the fifth leaf from the apex were collected since they do not show ontogenic resistance (age-related resistance) [69]. They were washed in distilled water and dried with a paper tissue. 

### 4.2. Suspension Preparation

*P. viticola* suspension was prepared using naturally infected leaves from the part of the vineyard where chemical protection was not applied. They were soaked in distilled water and gently brushed to detach sporangia from the leaf surface and make a dense suspension. It was adjusted to the concentration of 2 × 10^5^ sporangia mL^−1^ with Neubauer cell counting chamber (hemocytometer).

### 4.3. Leaf Discs Inoculation and Incubation

A cork borer was used to punch out 3.00 cm diameter leaf lamina parts (discs) from the leaves avoiding main veins. There were 24 leaf discs per genotype, half of which were inoculated with *P. viticola* suspension, while the other half was sprayed with distilled water (mock-inoculated leaf discs). Four leaf discs were placed in a Petri dish with the abaxial side up on a wet filter paper. The Petri dishes were sealed with parafilm and placed in a climate chamber (air temperature 20 °C, air moisture 80%). The samples were kept in dark for the first 24 h, while for the next seven days of incubation, a photoperiod of 16 h was applied. After 24 h drops of suspension and distilled water were collected with filter paper to avoid decaying of the leaf discs [22]. On the seventh day upon inoculation, the leaf discs were evaluated by ascribing to each one a percentage of the area covered by *P. viticola* fructification [70]. Finally, the average percentage of sporulation on the set of 12 inoculated leaf discs per genotype was scored according to the OIV descriptor 452-1 (Leaf: degree of resistance to *Plasmopara* (leaf disc test)) (Table 1 and Table 2) [30].

### 4.4. Chlorophyll Fluorescence and Multispectral Imaging

Chlorophyll fluorescence and multispectral imaging were carried out using the CropReporter^TM^ (PhenoVation B.V., Wageningen, the Netherlands). The measurements were performed seven times starting with no treated leaf discs and terminating with visible downy mildew symptoms (sporulation as white fuzz) on leaf discs’ abaxial side. Time points of imaging were as follows: before treatment (T_0_), one day post inoculation (dpi) (T_1_), two dpi (T_2_), three dpi (T_3_), four dpi (T_4_), six dpi (T_5_), and eight dpi (T_6_). Obtained parameters are summarised in Table 4. Leaf discs were imaged at a 45 cm distance from the camera always with the abaxial side up. The output is 16-bit RAW format. Automatic analysis of chlorophyll fluorescence, colour, and multispectral images was performed by DA^TM^ software (PhenoVation B.V., Wageningen, the Netherlands). The analysis was performed using regions of interest (the inner part of leaf discs) to avoid information of excised and senescing leaf disc’s edge [40]. 

Leaf discs were imaged with dark-to-light slow fluorescence induction [71], which includes dark adaptation, measurement of the induction curve of the dark-adapted leaf discs, followed by actinic light switching on for light adaptation, and measurement of induction curve of light-adapted leaf discs. For chlorophyll fluorescence measurements of dark-adapted leaf discs (30 min in dark before measurement), saturating light pulse (4500 μmol m^−2^ s^−1^ for 800 ms) was used. Minimum chlorophyll fluorescence (F_0_) was measured after 20 μs, and maximum chlorophyll fluorescence (F_m_) was measured after saturation. Four dark frames were captured and averaged to one single frame during the time red LEDs were off; overall, 20 frames were captured for the induction curve during 800 ms, and integration time for capturing the chlorophyll fluorescence images was 200 μs. 

After the measurement of dark-adapted leaf discs, they were relaxed in the dark for 15 s, and then actinic lights (300 μmol m^−2^ s^−1^) were switched on enabling leaf discs to adapt to light for 5 min. Steady-state fluorescence yield (F_s’_) was measured at the onset of the saturating pulse, and maximum chlorophyll fluorescence (F_m’_) of light-adapted plants was measured at saturation, using the saturating pulse intensity (4500 μmol m^−2^ s^−1^). Again, four dark frames were captured and averaged to one single frame during the time red LEDs were off; a total of 20 frames were captured for the induction curve during 800 ms, while integration time for capturing the chlorophyll fluorescence images was 200 μs.

Measured F_0_, F_m_, F_m’_, F_s’_ were used for calculation of the following fluorescence parameters, which include the following:

Maximum quantum yield of PSII (F_v_/F_m_): F_v_/F_m_ = (F_m_ − F_0_)/F_m_ [72];
Effective quantum yield of PSII (F_q’_/ F_m’_): F_q’_/ F_m’_ = (F_m’_ − F_s’_)/F_m’_ [72];
Electron transport rate (ETR) = F_q’_/F_m’_ × PPFD × (0.5) [72];
Non-photochemical quenching (NPQ) = (F_m_ − F_m’_)/F_m’_ [73].

Colour and spectral reflectance (R) images were captured after chlorophyll fluorescence imaging at 300 μmol m^−2^ s^−1^ produced by broadband white LEDs. Reflectance images were captured at Red—640 nm, Green—550 nm, Blue—475 nm, Chlorophyll (Chl)—730 nm, Anthocyanin (Anth)—540 nm, NIR—769 nm, and FarRed—710 nm.

From reflectance images, chlorophyll index (CHI) and anthocyanin index (ARI) were calculated using the following equations: CHI = (Chl)^−1^ − (NIR)^−1^ [74], and ARI = (Anth)^−1^ − (FarRed)^−1^ [75]. Hue was calculated after converting reflectance in Red, Green, and Blue into values between 0 and 1.

Hue (0–360°) was calculated as follows:

Hue = 60 × (0 + (Green − Blue)/(max − min)), if max = Red;
Hue = 60 × (2 + (Blue − Red)/(max − min)), if max = Green;
Hue = 60 × (4 + (Red − Green)/(max − min)), if max = Blue.
360 was added in the case of Hue < 0.

### 4.5. Statistical Analyses

Statistical analyses were performed by the XLSTAT statistical and data analysis solution (Addinsoft, 2020, New York, USA) [76]. The number of genotypes used in this study is large, and leaf discs are mostly excised from different leaves that provide heterogeneous samples. Subsequently, observations are contaminated with outliers, which was confirmed using an outlier test (data not shown). Thus, trimmed means are used for a better estimation of the most observations’ location. They are robust estimators of central tendency similar to the median [77]. To calculate a trimmed mean, a predetermined amount (25%) of observations of each side of the distribution of each genotype is removed and the remaining observations are averaged. 

Trimmed means are used for calculating logistic regression to find a relationship between the ascribed OIV classes and chlorophyll fluorescence parameters of leaf discs before *P. viticola* inoculation. The dependent variable (target) was the OIV classes (1, 3, 5, and 7) that are ascribed to each examined genotype according to the developed sporulation of downy mildew on leaf discs, while explanatory variables were the parameters of chlorophyll fluorescence and multispectral imaging summarised in Table 4. Since there are five categories (OIV classes) with the order, which are described in Table 2, an ordinal logistic regression and logit model with a confidence interval of 95% were used for the statistical analysis. The Newton–Raphson algorithm was used as a method of estimating the regression parameters. The OIV class 9 is considered completely resistant, and as such, was not included in this (modelling) part of the study.

Repeated measures ANOVA was performed to find differences in chlorophyll fluorescence and multispectral imaging parameters between infected and non-infected leaf discs and between infected leaf discs belonging to separated OIV classes throughout seven terms (from T_0_ to T_6_). The mean values, standard deviations, and significant differences of the data were calculated using XLSTAT (Addinsoft, New York, USA). The results were analysed using one-way ANOVA and the differences between the means were evaluated by Duncan’s multiple range test at a confidence level of 95% (*p* < 0.05).

## 5. Conclusions

The application of the leaf disc test proved to be an appropriate method for distinguishing grapevine genotypes according to their susceptibility to downy mildew. From a physiological point of view, chlorophyll fluorescence and multispectral imaging is a promising tool for precise monitoring of the photosynthesis transmission inside a leaf tissue upon *P. viticola* inoculation, as confirmed previously. Here, this utility is extended in a form of a possible phenotyping method among distinctive classes of grapevine genotypes in susceptibility to downy mildew in the absence of the pathogen. However, it is necessary to conduct more extensive experiments on a large number of genotypes, including the whole leaves and/or other susceptible tissues imaging. Certainly, there are morphological specificities in some cultivars (e.g., dense hydrophobic trichomes on the abaxial leaf sides) that act as a physical barrier and therefore cause lower susceptibility to downy mildew. Further research should also address scrutinised chemical analyses of grapevines’ secondary metabolites, such as polyphenolic and volatile compounds since their metabolomic pathways change upon pathogen’s attack and that feature could be peculiar to genotypes with a similar response to oomycetes.

## Figures and Tables

**Table 1:** Genotypes and their corresponding OIV classes 1, 3, 5, 7, and 9 from the most abundant to the totally absent sporulation.

Genotype	OIV Class
Babić	3
Belina starohrvatska	1
Belina svetokriška	5
Cabernet Sauvignon	5
Chardonnay	3
Crljenak viški	3
Debit	1
Divjaka	5
Dišeća ranina	5
Grk	1
Kadarun	5
Kraljevina	3
Lasina	1
Malvasija dubrovačka	3
Malvazija istarska	5
Mladenka	3
Moslavac	1
Ninčuša	3
Plavac mali	1
Plavčina	1
Plavina	1
Pošip	3
Regent	7
Solaris	7
Škrlet	3
Teran	5
Tribidrag	3
*V. riparia*	9
Žlahtina	5
Žumić	5

**Table 2:** The OIV 452-1 descriptor with images of visible differences between genotypes.

Representative Leaf Disc	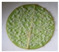	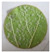	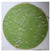	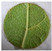	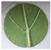
Genotype	Plavac mali	Babić	Malvazija istarska	Solaris	*V. riparia*
OIV class	1	3	5	7	9
Surface covered with sporulation (%)	61–100	41–60	21–40	1–20	0
Number of genotypes belonging to the class	8	10	9	2	1
Distribution of evaluated genotypes (%)	27	33	30	7	3

**Table 3:** Predicted OIV classes for ten native varieties in comparison with visual scoring. (1—variety belongs to this class by prediction; **1**—prediction matches with visual scoring).

		Predicted OIV Classes				
Variety	OIV Class by Visual Scoring	1	3	5	7	Prediction Correctness (%)
Plavčina	1	0	1	0	0	50
Plavina		**1**	0	0	0	
Moslavac		0	1	0	0	
Plavac mali		**1**	0	0	0	
Škrlet	3	0	**1**	0	0	50
Tribidrag		1	0	0	0	
Mladenka		0	0	1	0	
Ninčuša		0	**1**	0	0	
Belina svetokriška	5	0	0	**1**	0	100
Kadarun		0	0	**1**	0	

**Table 4:** Chlorophyll fluorescence and multispectral imaging parameters.

Parameter	Parameter Explanation
F_v_/F_m_	Maximum quantum yield of photosystem II (PSII) electron transport (leaf discs preconditioned in the dark)
F_q’_/F_m’_	Effective quantum yield of photosystem II (PSII) electron transport (leaf discs exposed to actinic light)
ETR	Electron transport rate
NPQ	Non-photochemical quenching (thermal energy dissipation in the PSII antennae)
qP	Photochemical quenching (proportion of open PSII reaction centres)
Hue	Indicator of colour differences (proportional to total chlorophyll content), colour appearance parameter
Far Red	Far-red reflectance
NIR	Near-infrared reflectance
CHI	Chlorophyll index
ARI	Anthocyanin reflection index
NDVI	Normalised difference vegetation index

## Data Availability

Data sharing not applicable.

## Abbreviations

OIV	International Organisation of Vine and Wine
OIV classes	1, 3, 5, 7, and 9 from the most susceptible to the completely resistant group of genotypes
T_0_–T_6_	terms of imaging from the pre-infection stage until the appearance of visible symptoms
dpi	day(s) post-inoculation
PSII	photosystem II
F_v_/F_m_	maximum quantum yield of photosystem II electron transport (variable to maximum value of chlorophyll *a* fluorescence)
F_q’_/F_m’_	effective quantum yield of photosystem II electron transport
ETR	electron transport rate
NPQ	non-photochemical quenching
qP	photochemical quenching
Hue	colour appearance parameter
Far Red	far red reflectance
NIR	near-infrared reflectance
CHI	chlorophyll index
ARI	anthocyanin reflection index
NDVI	normalised difference vegetation index
